# Differential Effects of p38, MAPK, PI3K or Rho Kinase Inhibitors on Bacterial Phagocytosis and Efferocytosis by Macrophages in COPD

**DOI:** 10.1371/journal.pone.0163139

**Published:** 2016-09-28

**Authors:** Martin A. Bewley, Kylie B. R. Belchamber, Kirandeep K. Chana, Richard C. Budd, Gavin Donaldson, Jadwiga A. Wedzicha, Christopher E. Brightling, Iain Kilty, Louise E. Donnelly, Peter J. Barnes, Dave Singh, Moira K. B. Whyte, David H. Dockrell, COPDMAP

**Affiliations:** 1 Department of Infection, Immunity and Cardiovascular Disease and The Florey Institute for Host-Pathogen Interactions, University of Sheffield Medical School, Sheffield, United Kingdom; 2 Airway Disease National Heart and Lung Institute, Imperial College London, London, United Kingdom; 3 Sheffield Teaching Hospitals Foundation Trust, Sheffield, United Kingdom; 4 Institute for Lung Heath, University of Leicester, Leicester, United Kingdom; 5 Pfizer Inc, Cambridge, Massachusetts, United States of America; 6 Centre for Respiratory Medicine and Allergy, Institute of Inflammation and Repair, Manchester Academic Health Science Centre, The University of Manchester and University Hospital of South Manchester NHS Foundation Trust, Manchester, United Kingdom; 7 Department of Respiratory Medicine and MRC Centre for Inflammation Research, University of Edinburgh, Edinburgh, United Kingdom; Medizinische Hochschule Hannover, GERMANY

## Abstract

Pulmonary inflammation and bacterial colonization are central to the pathogenesis of chronic obstructive pulmonary disease (COPD). Defects in macrophage phagocytosis of both bacteria and apoptotic cells contribute to the COPD phenotype. Small molecule inhibitors with anti-inflammatory activity against p38 mitogen activated protein kinases (MAPKs), phosphatidyl-inositol-3 kinase (PI3K) and Rho kinase (ROCK) are being investigated as novel therapeutics in COPD. Concerns exist, however, about off-target effects. We investigated the effect of p38 MAPK inhibitors (VX745 and SCIO469), specific inhibitors of PI3K α (NVS-P13K-2), δ (NVS-P13K-3) or γ (NVS-P13K-5) and a ROCK inhibitor PF4950834 on macrophage phagocytosis, early intracellular killing of bacteria and efferocytosis of apoptotic neutrophils. Alveolar macrophages (AM) obtained from broncho-alveolar lavage (BAL) or monocyte-derived macrophages (MDM) from COPD patients (GOLD stage II/III) enrolled from a well characterized clinical cohort (MRC COPD-MAP consortium) or from healthy ex-smoker controls were studied. Both COPD AM and MDM exhibited lower levels of bacterial phagocytosis (using *Streptococcus pneumoniae* and non-typeable *Haemophilus influenzae)* and efferocytosis than healthy controls. None of the inhibitors altered bacterial internalization or early intracellular bacterial killing in AM or MDM. Conversely PF4950834, but not other inhibitors, enhanced efferocytosis in COPD AM and MDM. These results suggest none of these inhibitors are likely to exacerbate phagocytosis-related defects in COPD, while confirming ROCK inhibitors can enhance efferocytosis in COPD.

## Introduction

Chronic obstructive pulmonary disease (COPD) is a chronic inflammatory lung condition in which patients suffer progressive worsening of lung function characterised by an obstructive pattern of airflow limitation, which is only partially reversible [1, 2]. Smoking is the main aetiological cause of the disease, which is expected to become the third leading cause of death worldwide by 2020 [3]. Patients with COPD have an accelerated decline in lung function and experience episodes of acute exacerbations, associated with increased lung inflammation. These events are a common cause of hospitalization [4, 5] and impose a considerable financial burden on health services.

Use of inhaled corticosteroids (ICS) in COPD patients reduces the rate of exacerbations, retards the rate of decline in quality of life measures and in the TORCH trial also the rate of decline in the forced expiratory volume in one second (FEV_1_); however, side effects of their use include an increased rate of pneumonia [6, 7]. The increased incidence of pneumonia in COPD itself and with ICS use emphasises the importance of developing alternative treatment strategies that do not further exacerbate the altered innate immune responses observed in COPD. In addition corticosteroids only partially block the induction of inflammatory cytokines in *ex vivo* experiments [8, 9]. There is, therefore, an unmet clinical need for the development of alternative anti-inflammatory therapies that do not significantly alter host defense, while ensuring a high-degree of modulation of pro-inflammatory responses. The human kinome contains multiple drugable targets which could be used to modify chronic inflammatory processes [10]. A variety of small molecule kinase inhibitors are being investigated as novel therapeutics with which to treat airway inflammation[11] and several have potential therapeutic value in COPD [12] [13].

The p38 mitogen-activated protein kinase (p38 MAPK) pathway stimulates pro-inflammatory cytokine expression [14], is activated by cigarette smoke [15] and has increased activity in COPD alveolar macrophages [16], a cell type in which p38 MAPK inhibition reduces cytokine expression [17]. An oral p38 inhibitor PH-797804 reached phase II clinical trials, with patients with moderate to severe COPD displaying improvements in lung function and dyspnoea over placebo [18]. Inhaled p38 inhibitors also have potential and may represent a means to limit systemic side-effects of treatment [19, 20] and are also in clinical trials for COPD (ClinicalTrials.gov identifier: NCT00642148) and could limit systemic side-effects of treatment. Phosphatidylinositol 3-kinases (PI3Ks) also showed increased activity in COPD patients [21]. Of the three isoforms of PI3K (α, γ and δ), PI3K γ is known to be pro-inflammatory and involved in neutrophil migration [22, 23] whereas PI3K δ activation contributes to corticosteroid resistance [21]. Furthermore, in a murine model of COPD, administration of an aerosolized PI3K γ/ δ inhibitor (TG100-115) suppressed lung inflammation [24]. Although PI3Kγ inhibitors are not in clinical development, PI3Kα inhibitors are in clinical trials for cancer and an oral PI3Kδ inhibitor (CAL-101) is in a Phase II trial for haematological malignancies (ClinicalTrials.gov identifier: NCT00710528). The Rho-associated protein kinase (ROCK) pathway is also implicated in the pathogenesis of COPD; ROCK is activated in the endothelial cells of smokers [25] and the pathway plays a role in the remodelling of the COPD airway, inducing contraction of airway smooth muscle [26]. ROCK also regulates the organization of stress fibres in fibroblasts, which contributes to fibrosis [27]. In addition ROCK pathways are also involved in the migration of inflammatory leukocytes [28] and ROCK inhibition has an anti-inflammatory effect on airways [29].

However, in addition to their pro-inflammatory function, p38 MAPK, PI3K and ROCK are known to have other roles within cells, including in the regulation of phagocytosis, efferocytosis and membrane trafficking [30–34]. To date, few studies have addressed how inhibition of these pathways might influence innate immune function of relevance to COPD. The lungs of patients with COPD are frequently colonized with bacteria, particularly *Haemophilus influenzae* and *Streptococcus pneumoniae* [35], and this colonization is associated with increased exacerbation frequency [36]. Alveolar macrophages play a critical role in the clearance of bacteria and apoptotic cells [37]. Any potential anti-inflammatory benefits of p38 MAPK, PI3K and ROCK inhibition would be tempered if there were also significant inhibition of key innate immune functions. We therefore tested whether p38, PI3K or ROCK inhibitors altered the ability of COPD macrophages to ingest bacteria and apoptotic cells using samples from a well-characterised clinical cohort of patients with COPD.

## Materials and Methods

### Macrophage donors

The COPD patients were enrolled in the UK Medical Research Council (MRC) COPD-MAP consortium of GOLD II/III patients. They were current or ex-smokers with a greater than 10 pack years smoking history and moderate to severe disease (GOLD stages II and III), (Tables 1 and 2). The controls were healthy current or ex-smokers smokers demonstrated not to have COPD after screening, which involved spirometry, clinical examination and a medical history. Exacerbation frequency was determined by questionnaire and frequent exacerbators were defined as having ≥ 2 exacerbations in the preceding 12 months. Alveolar macrophages (AM) and monocyte-derived macrophages (MDM) from COPD patients or healthy controls for this study were isolated from broncho-alveolar lavage (BAL) or whole blood respectively, with written informed consent. The study was approved by the National Research Ethics Service Committee of Yorkshire and the Humber, the National Research Ethics Service Committee of Manchester, and the National Research Ethics Service Committee of London.

**Table 1:** Demographics of participants in AM experiments.

	Healthy	COPD
**N**	12	16
**Age (years)**	58 ± 2	68 ± 2 *
**Gender M:F**	10:2	14:2
**Smoking History (Pack Years)**	10 ± 4	59 ± 8***
**FEV**_**1**_**(L)**	2.71 ± 0.2	1.90±0.2***
**FVC (L)**	3.66 ± 0.2	3.6 ± 0.2
**FEV**_**1**_ **(% predicted)**	109 ± 6	64 ± 3***
**FEV**_**1**_**:FVC**	0.74 ± 0.01	0.52 ± 0.02***
**GOLD Stage (II/III)**		16/0
**Non Frequent (NF)/Frequent Exacerbators (F)**		NF 12/F4

Data are presented as absolute numbers or mean ± SEM where

* represents p<0.05 and

*** p<0.001 for differences from healthy, comparison by unpaired t-test or Fisher’s Exact test.

**Table 2:** Demographics of participants in MDM experiments.

	Healthy	COPD
**N**	14	13
**Age (years)**	58 ± 2	71.8 ± 2 ***
**Gender M:F**	8:6	7:6
**Smoking History (Pack Years)**	11 ± 5	44 ± 10*
**FEV**_**1**_**(L)**	2.8 ± 0.2	1.3±0.1***
**FVC (L)**	3.8 ± 0.2	2.6 ± 0.2**
**FEV**_**1**_ **(% predicted)**	101.7 ± 3	72.7 ± 6**
**FEV**_**1**_**:FVC**	0.75 ± 0.02	0.49 ± 5.5***
**GOLD Stage (II/III)**		3/6

Data are presented as absolute numbers or mean ± SEM where

* represents p<0.05

** p<0.01 and

*** p<0.001 for differences from healthy, comparison by unpaired t-test or Fisher’s Exact test.

### Compounds

The p38 mitogen-activated protein kinase (MAPK) inhibitors used were SCIO469 or VX745 (Tocris). SCIO469 has a reported *in vitro* IC_50_ for p38α of 9nM with a 10-fold selectivity over p38β and >2000 fold selectivity over other kinases [38]. VX745 is also ~20-fold more selective for p38α over p38β with a reported Ki of 220nM and no activity against a panel of 50 kinases at 2μM [39]. The phosphatidylinositol-3-kinase (PI3K) class I isoform selective inhibitors NVS-PI3-2 (α selective), NVS-PI3-3 (δ selective) and NVS-PI3-5 (γ selective) were obtained from Novartis. The Rho-associated protein kinase (ROCK) inhibitor PF4950834 was obtained from Pfizer. Compounds were added at least 2 h before bacterial challenge or exposure to apoptotic cells at the indicated concentrations, and were present for the duration of experiments. Establishing levels of necrosis and apoptosis of macrophages confirmed lack of toxicity after exposure to each inhibitor or vehicle (DMSO) control.

### Cells and infection

Alveolar macrophages were isolated from BAL as previously described [40]. Cells were >95% alveolar macrophages as assessed by Diff-Quick staining (Dade Behring) visualised by light microscopy (Leica DMRB 1000). Due to limitations in cell number, not all assays were performed on all donors. Human monocyte-derived macrophages (MDM) were differentiated for 12 d from PBMC isolated from whole blood by Percoll (Sigma) gradient. Cells were cultured in RPMI (Lonza) supplemented with 10% (v/v) fetal calf serum (FCS) with low lipopolysaccharide (LPS) (Lonza) and were differentiated from PBMC by culturing with 2ng/ml granulocyte macrophage-colony stimulating factor (GM-CSF) (R&D Systems Ltd). Bone marrow-derived macrophages (BMDM) were obtained by culturing marrow from mice in DMEM containing 10% fetal calf serum (FCS) with low LPS (HyClone, Thermo Scientific) and 10% L929 conditioned medium for 14 days [41]. For experiments with live bacteria, cells were infected at a multiplicity of infection of 10. Following bacterial challenge, extracellular bacteria where washed off after 4 h, and media replaced.

### Bacteria

Serotype 14 *S*. *pneumoniae* (NCTC11902) was used in infections as a serotype commonly causing infection in the COPD lung [42]. Stocks were grown as previously described [43]. Non-typeable *H*. *influenzae* (NCTC 1269) was cultured on chocolate agar overnight and then grown to OD 0.6 in brain heart infusion (BHI) (Oxoid) supplemented with 20% (v/v) FCS (Sigma), 20 μg/ml NAD (Sigma) and 10 μg/ml Heme (Sigma). Bacteria were not opsonized. Heat-killed (HK) bacteria were generated by incubation at 65°C for 10 min as described previously [44].

### Fluorescent labelling of HK bacteria

Bacterial cultures were centrifuged and the pellet resuspended in 1ml NaHCO_3_ buffer containing 10 μl of AlexaFluor 488 NHS ester (1 mg lyophilised dye in 1ml DMSO, Life Technologies) and incubated overnight at room temperature on a windmill rotator. The bacteria were then centrifuged and the supernatant aspirated before the pellet was resuspended in 1ml PBS and washed twice. The final pellet was resuspended in 1ml PBS. Samples were pooled together and the dilution adjusted to give an OD_600_ of between 1.8 and 2.0 for *S*. *pneumoniae* or 1.5–1.7 for *H*. *influenzae*. Samples were stored at -20°C

### Bacterial internalization

Viable intracellular bacteria were measured at 4 h post-challenge as a measure of bacterial internalization using a gentamicin protection assay as previously described [45]. At 4 h cells were washed three times in PBS before being incubated for 30 min at 37°C in RPMI containing 40 μU penicillin (Sigma) and 20 μg/ml gentamicin (Sanofi) to kill extracellular bacteria. Cells were then washed three times in PBS before being lysed in 250 μl 2% (w/v) saponin for 12 min. The lysate was made to 1 ml in PBS, and intracellular bacterial numbers determined by Miles-Misra viable count. To measure internalisation of fluorescently labelled HK bacteria, fluorescent stocks (as above) were sonicated to ensure even distribution of bacteria. 100 μL of bacterial stock was added to 900 μL RPMI. Media was removed from cells and 100μL of bacterial suspension was added and incubated at 37°C for 4 h. Cells were then washed and extracellular fluorescence quenched by adding 100 μL trypan blue (Sigma, 2% v/v) for 2 min at room temperature. The trypan blue was then discarded and fluorescence determined using a fluorometric plate reader (BMG Fluorostar) excitation 480nm, emission 520nm.

### Bacterial killing

For assessment of early *S*. *pneumoniae* killing, cells were infected for 2 h before extracellular bacteria were killed at 2 h as in the internalization experiment above, after which cultures were placed in media containing 0.75μg/ml vancomycin. At the designated time points, levels of intracellular viable bacteria were then determined as above.

### Efferocytosis

Neutrophils from whole blood were purified by Percoll gradient, before being washed in serum free media. Neutrophils were stained with PKH-26 red fluorescent dye (Sigma) according to manufacturer’s instructions. Staining was stopped by addition of 2ml 1% (w/v) BSA. Cells were washed three times in RPMI before being resuspended at 5.0 x 10^6^ cells/ml in RPMI supplemented with 10% (v/v) FCS. Cells were then cultured for 20 h. This resulted in >80% apoptotic and fewer than 5% necrotic cells as measured by Annexin V Topro staining [46]. Apoptotic cells were added to macrophages at an MOI of 5 for a period of 90 min. Cells were then washed to remove non internalized neutrophils, and fluorescence analysed by flow cytometry using a BD FacsCalibur and the FL-2 channel. 10,000 events were captured and median fluorescence intensity (MFI) was recorded. The MFI of duplicate samples incubated on ice for 90min were subtracted to control for surface-bound non-internalized neutrophils.

### Toxicity assays

Nuclear fragmentation and condensation indicative of apoptosis were detected at 20 h using 4′6′-diamidino-2-phenylindole (DAPI), by reviewers blinded to sample origin, as previously described [43]. Necrosis was measured by the release of lactate dehydrogenase into supernatants using the Cytotox 96 cell viability kit (Promega), used according to the manufacturer’s instructions. Alternatively, cell viability was measured using metabolic activity as a surrogate, using 0.1% (w/v) MTT-3-(4,5-Dimethylthiazol-2-yl)-2,5-diphenyltetrazolium bromide (MTT). Cells were incubated with 50μl MTT per well at 37°C for 30 min. MTT was removed and 50μl of dimethyl sulfoxide (DMSO) added to lyse the cells. Absorbance was read using a Spectramax photometer at 570nm and values normalized to 100% non-stimulated cell controls for each condition.

### Western blot

Whole cell extracts were isolated using SDS-lysis buffer as described before [47] and equal protein loaded per lane. Proteins were separated by SDS gel electrophoresis, blotted onto a PVDF membrane, and blocked for 60 min at room temperature in PBS containing 0.05% (v/v) Tween with 5% (w/v) skim milk powder. Membranes were incubated overnight at 4°C with antibodies against either heat-shock protein 27 (HSP27) (HSP27(G31), Mouse monoclonal #2402, Cell Signalling 1:1000), phospho-HSP27 (p-HSP27(Ser82), rabbit monoclonal #2401, Cell signalling, 1:1000), protein kinase B (AKT) (rabbit polyclonal #9271, Cell signalling 1:1000), phospho-AKT (p-AKT) (p-AKT(Ser473), rabbit polyclonal #9271, Cell signalling 1:1000), myosin light chain 2 (MLC) (rabbit polyclonal #3672, Cell signalling, 1:1000), or phosphorylated-MLC (p-MLC) (p-MLC(Thr18/ser19), rabbit polyclonal #3672, Cell signalling, 1:1000). Proteins were detected using HRP-conjugated secondary antibodies (1:2000; Dako) and ECL (Amersham Pharmacia).

### Cytokine production

COPD AM cultures were pre-treated with compounds for 2 hours, before being stimulated with S14 at an MOI of 10. Supernatants harvested after 20 h. TNF-α and IL-6 levels were measured by ELISA (R & D Systems) according to manufacturer's instructions.

### Statistics

Data are presented as medians or means with error bars indicating +/- SEM or IQR. Comparisons were made by Student’s t-test or Mann-Whitney U, or if the data were matched, by paired t-test or Wilcoxon signed rank test. Friedman’s test was used to make comparisons involving repeated measures. Significance was defined as *P* < 0.05. Statistical tests were performed using Prism 6.0 software (GraphPad Inc.).

## Results

### Demographic data

The characteristics of all of the macrophage donors are described in Tables 1 and 2. Alveolar macrophage donors with COPD (Table 1) and MDM donors with COPD (Table 2) had a significantly greater number of pack years of cigarette exposure, and had significantly lower FEV_1_ (L), % predicted FEV_1_ and FEV_1_:FVC ratio compared to the healthy controls. All samples were collected when the patient was clinically stable (free of acute exacerbation) and none of the patients were taking long term antibiotics.

### p38 MAPK, ROCK or PI3K inhibitors have low toxicity and are active in COPD alveolar macrophages

**Fig 1:**
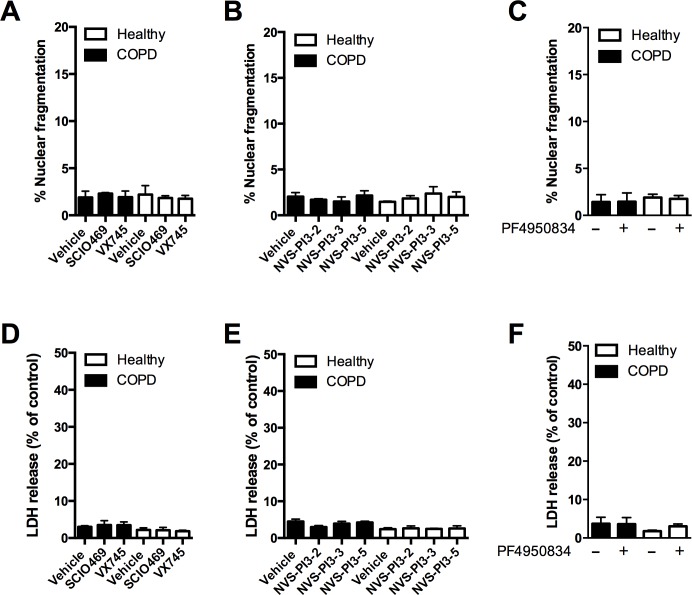
Cytotoxicity and efficacy of p38, PI3K and ROCK inhibition in macrophages. **(A-F)** Alveolar macrophages (AM) from COPD patients or healthy controls were incubated with either vehicle (-), or incubated with (+) 1μM SCIO469, 1μM VX745, 100nM NVS-PI3K-2/3/5 or 200nM PF4950834 for 20 h, before cultures were assessed for apoptosis (A-C) by nuclear fragmentation, or necrosis (D-F). In all experiments, n = 4, there was no significant differences between groups.

In order to establish that any modification of macrophage responses observed was not due to compounds affecting cell viability, alveolar macrophages from healthy donors or COPD patients were assessed for apoptosis (Fig 1A–1C) or necrosis (Fig 1D–1F) after treatment with p38 inhibitors SCIO469 and VX745 (Fig 1A and 1D), PI3K inhibitors NVS-PI3-2, NVS-PI3-3 or NVS-PI3-5 (Fig 1B and 1E) or the ROCK inhibitor PF4950834 (Fig 1C and 1F) for 20 h. None of the compounds induced either apoptosis or necrosis compared to vehicle controls.

**Fig 2:**
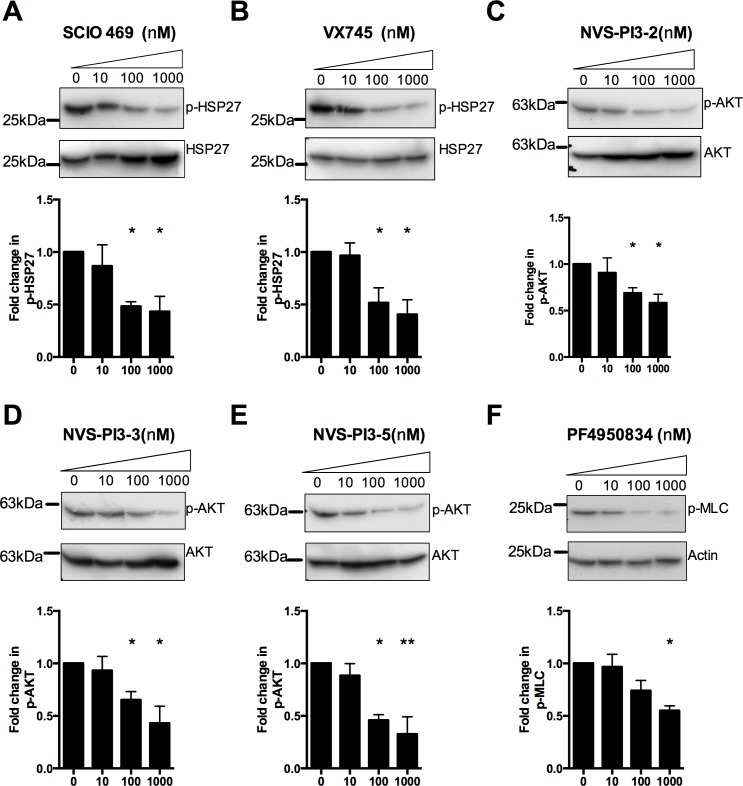
p38, PI3K and ROCK inhibition modulates signalling in alveolar macrophages. **(A-F)** COPD alveolar macrophages (AM) were pre-treated with the designated concentrations of SCIO469 (A) VX745 (B), NVS-PI3K-2/3/5 (C-E), or PF4950834 (F), were then challenged with *S*. *pneumoniae* for 6 h, before cells were lysed and probed for either p-HSP27 (A-B), p-AKT (C-E), or p-MLC (F). Plots are representative of three independent experiments and densitometry from all three experiments are shown, * = p<0.05, ** = p<0.01, ANOVA with Dunnetts post-test vs control.

**Fig 3:**
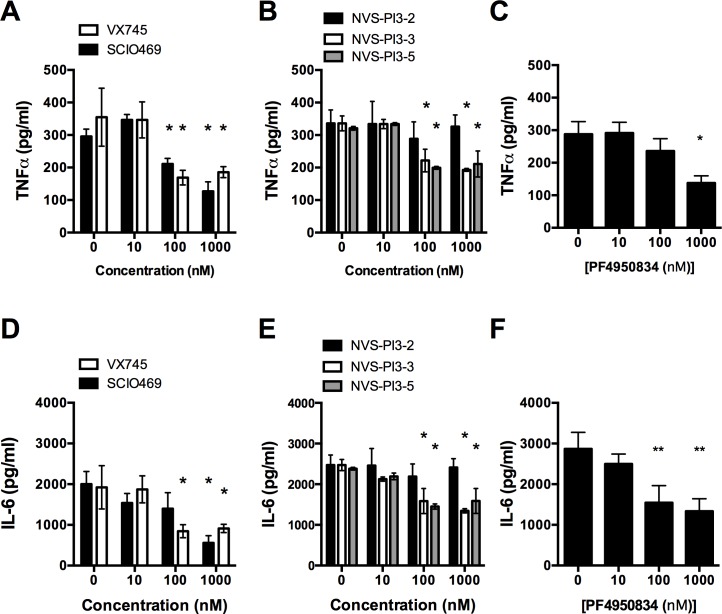
p38, PI3K and ROCK inhibition modulates cytokine production in alveolar macrophages. **(A-F)** COPD alveolar macrophages (AM) were pre-treated with the designated concentrations of SCIO469 or VX745 (A and D), NVS-PI3K-2/3/5 (B and E), or PF4950834 (C and F), before challenge with *S*. *pneumoniae* for 6 h. Supernatants were collected and levels of TNFα (A-C) and IL-6 (D-F) were measured by ELISA, n = 4, * = p<0.05, ANOVA with Dunnetts post-test vs control.

To ensure that the compounds were functional at the concentrations administered, in COPD alveolar macrophages, their ability to inhibit their respective pathways was assessed. Treatment of alveolar macrophages with SCIO469 and VX745 resulted in a decrease in the inducible levels following bacterial challenge of the activated, phosphorylated form of pHSP27 a downstream target of MAPK signalling [48] (Fig 2A and 1B). Similarly, NVS-PI3-2, NVS-PI3-3 or NVS-PI3-5 treatment reduced AKT phosphorylation after bacterial challenge, a downstream event in PI3K signalling [49] (Fig 2C–2E). Treatment of cells with PF4950834 resulted in a decrease in the level of phosphorylated regulatory light chain of Myosin II/Mysosin light chain (p-MLC) after bacterial challenge, a downstream consequence of ROCK signalling [50] (Fig 2F). To further check the efficacy of the compounds we next looked at their ability to modulate pro-inflammatory cytokine production from alveolar macrophages. Inhibition of p38 with VX745 significantly reduced TNFα and IL-6 production at concentrations of 100nM and above, whereas treatment with SCIO469 inhibited TNFα and IL-6 at concentrations of 100nM and 1000nM respectively (Fig 3A and 3D). Similarly, inhibition of PI3Kα and P13Kδ with NVS-PI3-2 and NVS-PI3-3 significantly reduced TNFα and IL-6 production at concentrations of 100nM (Fig 3B and 3E). The role of PI3Kγ in macrophages has predominantly thought to be in regulating the chemotactic response [23, 51, 52], and inhibition of PI3Kγ did not affect cytokine production in our model (Fig 3B and 3E). In keeping with its role in cytokine production in macrophages [53], inhibition of ROCK (Fig 3C and 3F) significantly reduced TNF or IL-6 production, at doses from 1000nm and 100nm respectively. Thus treatment of macrophages with all classes of inhibitor resulted in anticipated changes in downstream signalling consistent with activity of the inhibitors and no adverse effects seen on cell viability were seen at the concentrations used.

### COPD alveolar macrophages show reduced phagocytosis of *S*. *pneumoniae* compared to healthy macrophages, and this is not affected by inhibition of p38, ROCK or PI3K pathways

**Fig 4:**
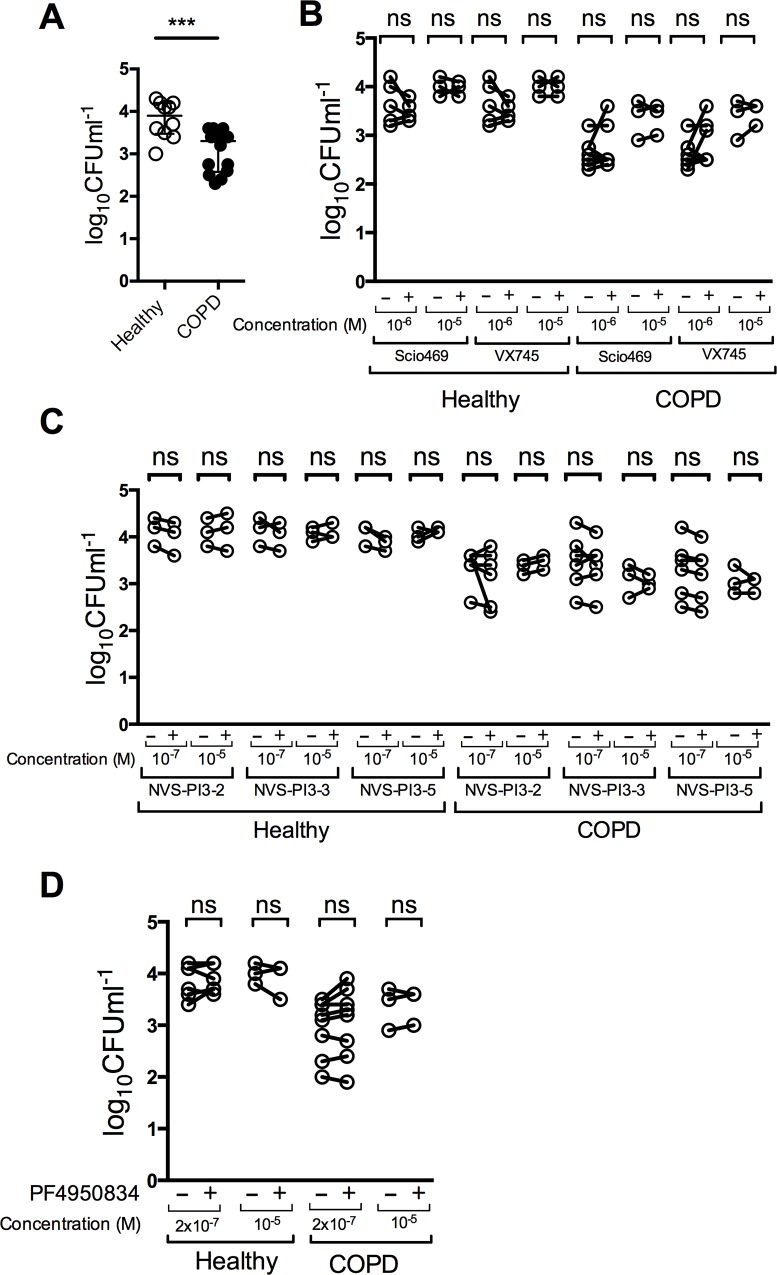
COPD alveolar macrophages have reduced phagocytosis of *S*. *pneumoniae*, which is not modified by p38, PI3K or ROCK inhibition. **(A)** Alveolar macrophages (AM) from COPD patients or healthy controls were challenged with *S*. *pneumoniae* (Spn) at a multiplicity of infection (MOI) of 10. 4 h post challenge, the number of viable intracellular bacteria was determined. Data presented as median ± IQR, n = 10/14 healthy/COPD, *** = p<0.001, Mann-Whitney U test. **(B-D)** Healthy or COPD AM were treated with vehicle (-) or the designated doses of SCIO469, VX745 (B), NVS-PI3K-2/3/5, (C) or PF4950834 (D) before challenge with Spn at MOI 10. 4 h post challenge, numbers of viable internalized bacteria were determined, n = 3–5, data shown as paired vehicle and compound data for each donor, ns = non-significant, paired t- test.

The ability of macrophages to phagocytose and kill bacteria is one of their key immune functions [37]. The p38 MAPK, PI3K and ROCK pathways have all been shown to modulate phagocytosis in various models [54–58]. We therefore investigated the effect of inhibiting p38, PI3K and ROCK pathways in alveolar macrophages on bacterial phagocytosis. Measurement of viable intracellular *S*. *pneumoniae* at 4 h after bacterial challenge revealed that COPD patients’ alveolar macrophages had lower numbers of intracellular viable bacteria than healthy controls (Fig 4A). Treatment of either healthy donor or COPD patients’ alveolar macrophages with p38 inhibitors SCIO469 and VX745 (Fig 4B), PI3K inhibitors NVS-PI3-2, NVS-PI3-3 or NVS-PI3-5 (Fig 4C) or the ROCK inhibitor PF4950834 (Fig 4D) resulted in similar levels of internalised bacteria when compared to the matched donor sample treated with vehicle controls.

**Fig 5:**
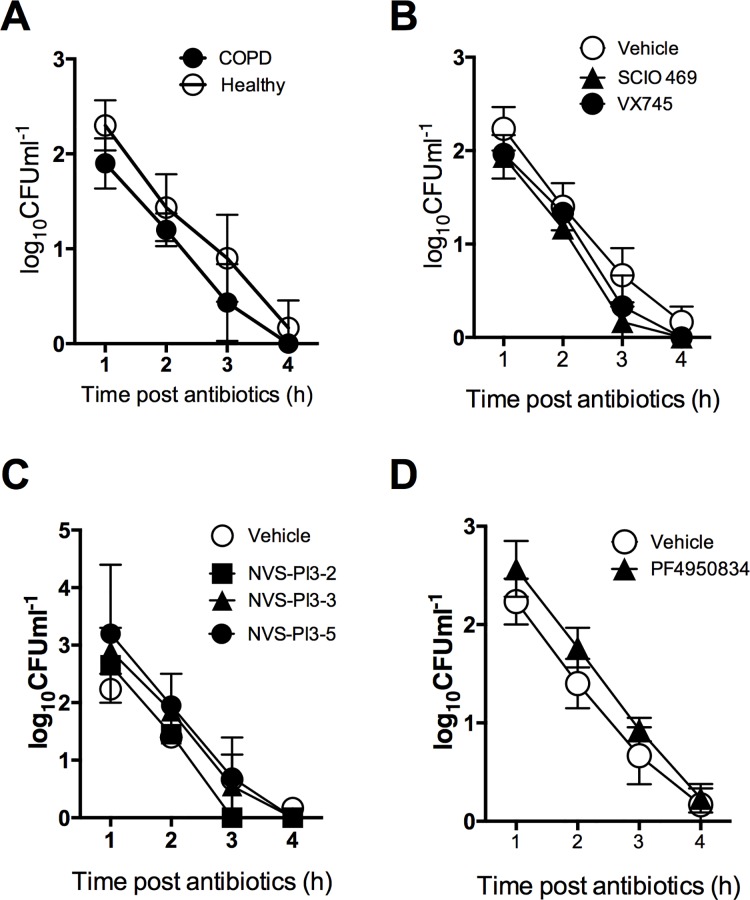
p38, PI3K or ROCK inhibition does not affect early-phase bacterial killing in alveolar macrophages. **(A)** Alveolar macrophages (AM) from healthy donors or COPD patients were challenged with *S*. *pneumoniae* (Spn) at a multiplicity of infection of 10. 2 h after challenge non-internalised bacteria were washed off, and antibiotics added. At the designated time post-antimicrobials persisting viable bacteria were measured. **(B-D)** COPD AM were pre-treated with vehicle or the designated inhibitor before being challenged with Spn at MOI of 10. At the designated time post-antimicrobials persisting viable bacteria were measured. In all experiments, n = 4, with no significant differences between any groups at any time point, Friedman test.

Since the number of viable intracellular bacteria is potentially influenced by both the phagocytosis of bacteria and the rate of early intracellular killing of *S*. *pneumoniae* in the phagolysosome [40] we performed an additional assay to measure the kinetics of early intracellular killing, after all extracellular bacteria were killed. Although the starting number of intracellular bacteria was higher in healthy AM than in the COPD patient’s alveolar macrophages, the rate of decay, as a measure of bactericidal killing, was similar between healthy and COPD donors (Fig 5A). This proved that the initial differences in intracellular viable bacteria between COPD and healthy donor alveolar macrophages were the result of differences in phagocytosis not early intracellular killing. Similarly, potential alterations in phagocytosis were not being masked by the compounds altering early bacterial killing as the rate of killing in COPD macrophages for cells treated with SCIO469 and VX745 (Fig 5B), PI3K inhibitors NVS-PI3-2, NVS-PI3-3 or NVS-PI3-5 (Fig 5C) or the ROCK inhibitor PF4950834 (Fig 5D), were not significantly different to the paired donor samples treated with vehicle control. These results suggested that although COPD patients’ alveolar macrophages were less efficient at phagocytosis of *S*. *pneumoniae* none of the compounds studied modified either phagocytosis or early intracellular killing of bacteria.

### COPD monocyte-derived macrophages show reduced phagocytosis of bacteria compared to healthy macrophages, and this is not affected by inhibition of p38, ROCK or PI3K pathways

**Fig 6:**
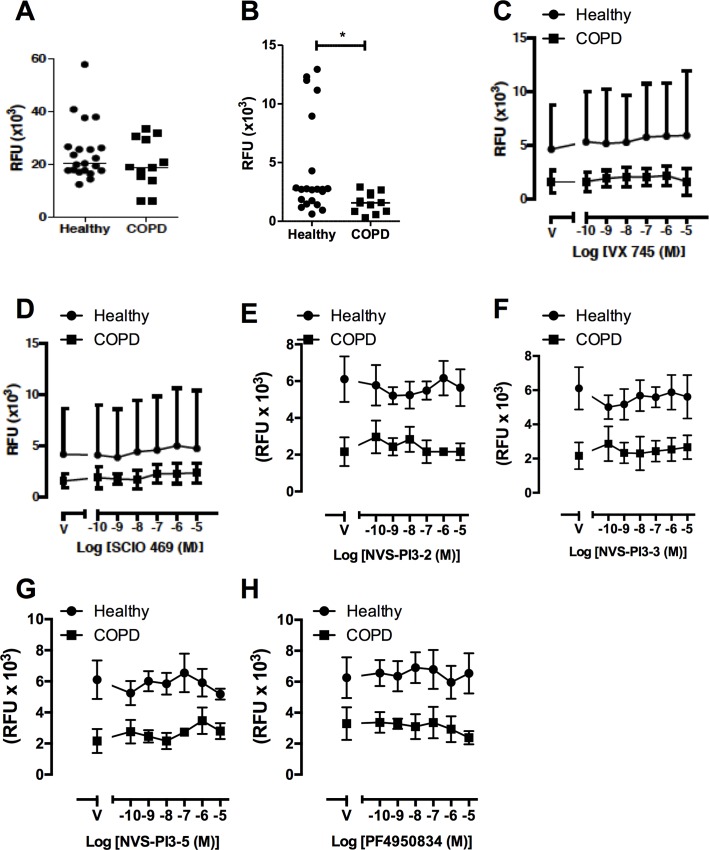
COPD MDM have reduced phagocytosis of *S*. *pneumoniae* which is not modified by p38, PI3K or ROCK inhibition. **(A-B)** MDM from healthy donors or patients with COPD were incubated with fluorescent beads (A) or fluorescently labelled *S*. *pneumoniae* (B) for 4h and phagocytosis measured by fluorimetry. Data are presented as individual data points and the line represents median *p<0.05 Mann-Whitney U test. **(C-D)** MDM from healthy donors or patients with COPD were pre-incubated with p38 inhibitors VX745 (C) or SCIO469 (B) for 1h prior to challenge with fluorescently labelled *S*. *pneumoniae* for 4h. Data are presented as mean ± SEM for n = 10 healthy donors and n = 6 COPD. **(E-G)** MDM from healthy donors or patients with COPD were pre-incubated with the PI3K inhibitors NVS-PI3-2 (E), NVS-PI3-3 (F) or NVS-PI3-5 (G) for 2h prior to challenge with fluorescently labelled *S*. *pneumoniae* for 4h. Data presented as mean ± SEM for n = 3 healthy and n = 3 COPD. **(H)** MDM from healthy donors or patients with COPD were pre-incubated for 2h with the ROCK inhibitor, PF4950834 prior to challenge with fluorescently labelled *S*. *pneumoniae* for 4h. Data are presented as mean ± SEM for n = 3 healthy and n = 3 COPD. In all experiments, no significant differences were observed in internalization.

**Fig 7:**
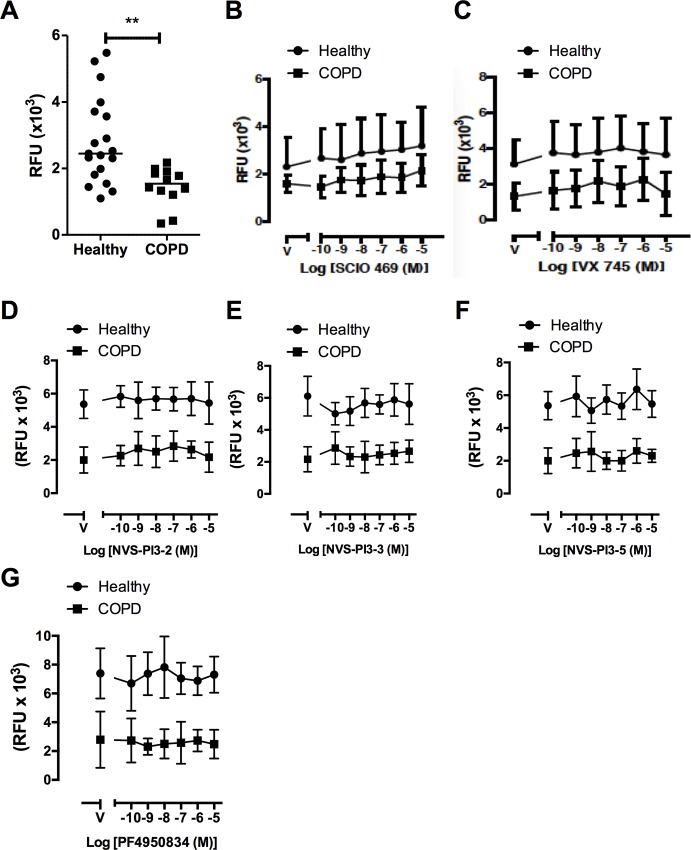
COPD MDM have reduced phagocytosis of *H*. *influenzae*, which is not modified by p38, PI3K or ROCK inhibition. **(A)** MDM from healthy donors or COPD patients were challenged with fluorescently labelled *H*. *influenzae* for 4h and phagocytosis measured by fluorimetry. Data are presented as individual data points and the line represents median where **p<0.01 Mann-Whitney U test. **(B-C)** MDM from healthy donors or patients with COPD were pre-incubated with p38 inhibitors VX745 (B) or SCIO469 (C) for 2h prior to challenge with fluorescently labelled *S*.*pneumoniae* for 4h. Data are presented as mean ± SEM for n = 10 healthy donors and n = 6 COPD. **(D-F)** MDM from healthy donors or patients with COPD were pre-incubated with the PI3K inhibitors NVS-PI3-2 (D), NVS-PI3-3 (E) or NVS-PI3-5 (F) for 2h prior to challenge with fluorescently labelled *S*.*pneumoniae* for 4h. Data presented as mean ± SEM for n = 3 healthy and n = 3 COPD. **(G)** MDM from healthy donors or patients with COPD were pre-incubated for 2h with the ROCK inhibitor, PF4950834 prior to challenge with fluorescently labelled *H*. *influenzae* for 4h. Data are presented as mean ± SEM for n = 3 healthy and n = 3 COPD. In all experiments, no significant differences were observed in internalisation between vehicle and any concentration of compound for either healthy or COPD MDM.

We have previously shown that COPD phagocytic defects extend beyond AM [44] and confirmed this finding by showing that although the ability of MDM to phagocytose beads was similar in cells from controls and COPD patients, uptake of *S*. *peumoniae* was suppressed (Fig 6A and 6B). Next we tested whether the kinase inhibitors studied in AM had any effect on phagocytosis of *S*. *pneumoniae* by MDM. MDM were treated with increasing concentrations of p38 inhibitors SCIO469 and VX745 (Fig 6C and 6D) the PI3K inhibitors NVS-PI3-2, NVS-PI3-3 or NVS-PI3-5 (Fig 6E–6G) and the ROCK inhibitor PF4950834 (Fig 6H), none of which significantly affected phagocytosis of *S*. *pneumoniae*. When phagocytosis of non-typeable *H*. *influenzae* was examined we also confirmed reduced phagocytosis in COPD MDM but failed to detect any reduction in phagocytosis in any group at any concentration with each of the kinase inhibitors (Fig 7). There was no effect on cell viability as tested by MTT assay with any of these kinase inhibitors at the range of doses used in these experiments (data not shown). Experiments with *S*. *pneumoniae* were also performed in murine bone-marrow derived macrophages. Results corroborated those in human cells, with no inhibition of phagocytosis seen for any compound (S1 Fig).

### Inhibition of the ROCK pathway enhances defective efferocytosis in COPD macrophages

**Fig 8:**
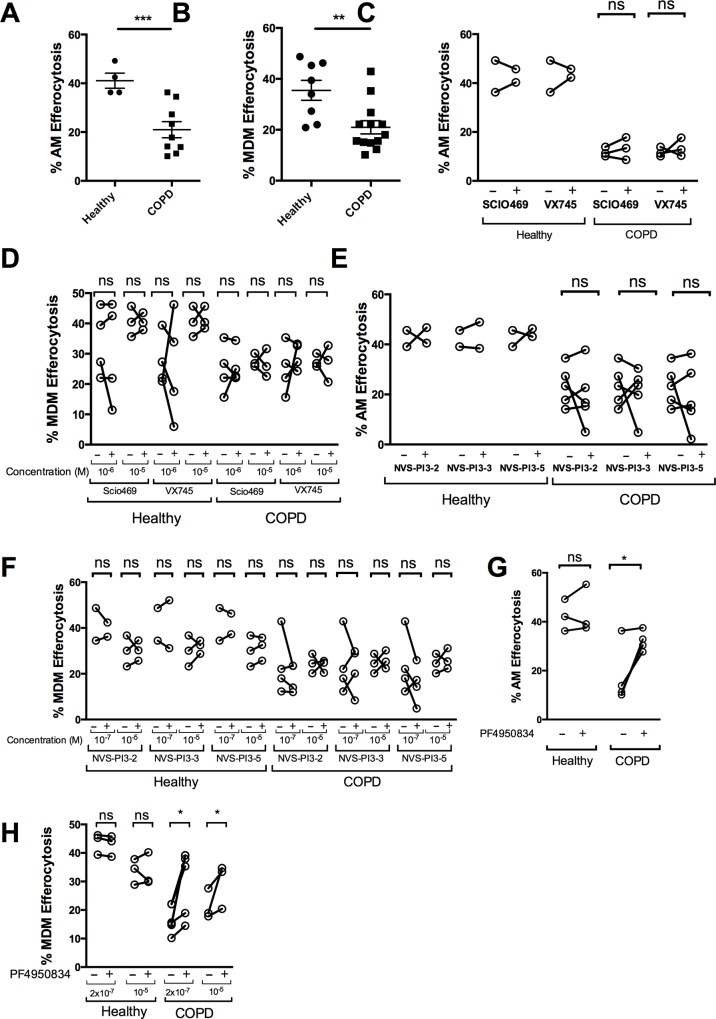
Inhibition of ROCK, but not p38 or PI3K pathways, increases efferocytosis in COPD alveolar and monocyte-derived macrophages. Alveolar (AM) or monocyte-derived macrophages (MDM) were incubated with PKH-26 stained apoptotic neutrophils for 90 min, before efferocytosis was assessed by flow cytometry. (**A-B)** Pooled vehicle data for AM, (A) n = 4–9, *** = p<0.001, Mann-Whitney and MDM (B), n = 7–12, ** = p<0.01, Student t-test. **(C-H)** Healthy or COPD AM (C, E and G) were pre-treated with vehicle (-) or 1μM SCIO469, 1μM VX745 (C), 100nM NVS-PI3K-2/3/5 (E), or 200nM PF4950834 (G) (+). MDM (D, F and H) were treated with vehicle (-) or compounds at the designated dose (+), ns = non significant, * = p<0.05, Wilcoxon matched pairs test.

Efferocytosis of apoptotic cells is also a critical function of macrophages, facilitating the removal of apoptotic material and resolution of inflammation [59–61]. Defective efferocytosis and the increased presence of apoptotic cells have been identified in the airways of subjects with COPD [62]. In keeping with this, COPD alveolar macrophages and MDM had lower rates of efferocytosis than healthy AM (Fig 8A and 8B). Treatment of healthy or COPD AM and MDM with either SCIO469 or VX745 (Fig 8C and 8D), or PI3K inhibitors NVS-PI3-2, NVS-PI3-3 or NVS-PI3-5 (Fig 8E and 8F) did not alter rates of efferocytosis. However, in COPD AM and MDM treatment with the ROCK inhibitor PF4950834 enhanced efferocytosis, although no uplift was seen for healthy AM or MDM (Fig 8G and 8H).

## Discussion

The central role of pulmonary inflammation in the pathogenesis of COPD is well established [63]. Multiple factors contribute to the persistent neutrophilic inflammation in the COPD lung, including activation of pattern recognition receptors by pathogen-associated molecular patterns (PAMPS) associated with colonizing bacteria, and damage-associated molecular patterns (DAMPS) generated by host cell death during infections and tissue remodelling. Oxidant stress contributes to the initiation and persistence of the pro-inflammatory state and genetic susceptibility and epigenetic reprogramming further amplifies inflammation [64, 65]. On-going inflammation is further accentuated by impaired clearance of bacteria and apoptotic cells from the lung [44, 62]. Strategies to alter pulmonary inflammation are therefore attractive, but must ensure that reduction of inflammation is not achieved at the cost of inhibiting antibacterial host defence or efferocytosis.

The quest for anti-inflammatory or disease modifying therapies for the treatment of COPD has led to investigation of a number of putative kinome targets. The most advanced target in drug development for the treatment of COPD is p38 MAPK with several clinical trials reporting modest effects of oral inhibitors on inflammatory readouts [66, 67]. Studies have shown efficacy of p38 inhibitors in COPD with improvement in lung function and dyspnoea scores over placebo [18]. We and others have, however, demonstrated that innate immune functions are attenuated in macrophages from COPD patients [44, 68]. Since the p38 pathway is active in monocytes and macrophages [69, 70] it was important to establish whether bacterial clearance or efferocytosis by macrophages was altered by p38 MAPK inhibition in order to establish if the therapeutic approach had unforeseen risks.

The present study clearly shows that inhibition of the p38 MAPK pathway, using two structurally distinct chemotypes, does not alter phagocytosis of bacteria, early bacterial killing of bacteria or efferocytosis by macrophages. These results corroborate other studies which show bacterial phagocytosis by neutrophils activates p38α but administration of a p38 MAPK inhibitor fails to attenuate phagocytosis [71] and that p38 MAPK inhibition failed to block murine bone marrow derived macrophage uptake of beads [72]. Conversely, TLR-mediated p38 activation has been shown to increase bacterial phagocytosis in murine and human macrophages through upregulation of scavenger receptors [73]. Similarly, the adenosine analogue 5-aminoimidazole-4-carboxamide-1-β-D-ribofuranoside (AICAR) has been shown to augment phagocytic pathways at least in part by a p38 MAPK mediated mechanism that could be suppressed by both small molecule inhibitors or targeted siRNA [74]. The discrepancy in these results is unlikely to reflect different kinase utilisation between human and murine models, as p38 inhibition did not impair phagocytosis in murine BMDM in our model (S1 Fig). Rather, the discrepancy likely reflects the complexity of phagocytosis and efferocytosis signalling pathways, the involvement of which is often dependent on cell type and stimuli used. It is therefore possible that additional stimuli are required to engage the previously described p38 MAPK pathways and that these are less important in the COPD lung. It also reflects the fact that our bacterial incubation period was relatively short so less likely to alter the TLR signalling, our read out of non-opsonic bacterial uptake was less likely to be influenced by TLR signalling and that COPD macrophages may be less able to alter their activation status to a more classical activation state [75].

Other kinases have also been implicated in COPD pathology including PI3K and inhibition of this protein has been shown to restore corticosteroid insensitivity [21], resulting in a high-level of interest in targeting this pathway. PI3K is known to be important in macrophage migration, for example towards the chemoattractant CCL2 [52], while PI3Kγ^-/-^ mice showed reduced neutrophil and macrophage chemotaxis to inflammatory stimuli [23]. The PI3K pathway is involved in the control of pseudopod formation [76], and is required for Fc gamma receptor (FcγR)-mediated phagocytosis in macrophages [56]. However, utilisation of PI3K pathways downstream of other phagocytic receptors is more multifaceted. Inhibition of PI3K prevents phagocytosis of zymosan [77] and in peritoneal macrophages suppresses phagocytosis of *Helicobacter pylori* through modification of actin polymerisation at sites of uptake [78]. However, inhibition of PI3K does not inhibit the phagocytosis of *Salmonella typhimurium* [79], *Legionella pneumophila* [80], or *Escherichia coli* [81], indicating that only a subset of phagocytic responses are regulated by PI3K. The present study shows that inhibition of individual subunits of PI3K, using three different inhibitors, does not alter phagocytosis or intracellular killing of *S*. *pneumoniae* or *H*. *influenzae* by human macrophages, again suggesting differential pathway regulation in these cells.

The ROCK pathway has been implicated in modulation of phagocytosis in macrophages [82], while inhibition of the pathway is anti-inflammatory in the airways [29]. Inhibition of ROCK reverses the reduction in efferocytosis induced by oxidant stress or alcohol exposure in AM [83] another setting associated with altered oxidative stress [84], indicating that ROCK inhibition could enhance efferocytosis under conditions similar to those in COPD. The present study shows that inhibition of ROCK, while not altering phagocytosis or intracellular killing of bacteria by human macrophages, causes an increase in efferocytosis of apoptotic neutrophils by alveolar macrophages from COPD patients, but not in healthy controls. This supports the hypothesis that inhibition of ROCK reverses the defect in efferocytosis specifically seen in the environment of the COPD lung and highlights that specific differences exist in the pathways that regulate phagocytosis of bacteria and efferocytosis in COPD, despite the fact that both processes are perturbed in the COPD lung.

Overall these results illustrate that inhibition of several distinct kinase pathways with the potential to adversely influence bacterial phagocytosis or efferocytosis have no such adverse effects on AM or MDM isolated from individuals with COPD. These findings are reassuring and suggest that inhibition of these pathways will not impact on these aspects of the suppressed innate immune responses of COPD macrophages. The ability to study AM isolated directly from the COPD lung increases the strength of these conclusions. Moreover, we found that a ROCK inhibitor partially corrects the COPD associated defect in AM efferocytosis with no adverse effect on bacterial phagocytosis. The strategy of targeting these pathways, as potential novel anti-inflammatory treatments, remains a potential therapeutic approach. These data add confidence that this approach will not have a negative consequence on bacterial clearance or removal of apoptotic bodies.

## Supporting Information

S1 Fig Inhibition of p38, PI3K or Rho kinases in murine bone-marrow derived macrophages (BMDM) does not affect phagocytosis of *S*. *pneumoniae*. BMDM were treated with vehicle (V) or the designated doses of SCIO469, VX745 (B), NVS-PI3K-2/3/5, (C) or PF4950834 (D) before challenge with Spn at MOI 10. 4 h post challenge, numbers of viable internalized bacteria were determined, n = 3, no significant differences between vehicle and any dose of compound.
(TIFF)

S2 Fig Full length Western blots from Fig 2. (TIFF)
