# Supplementary figures and images for: Differential Effects of p38, MAPK, PI3K or Rho Kinase Inhibitors on Bacterial Phagocytosis and Efferocytosis by Macrophages in COPD
Source: PLoS One. 2016 Sep 28;11(9):e0163139. doi: 10.1371/journal.pone.0163139 (PMC5040258; doi:10.1371/journal.pone.0163139)

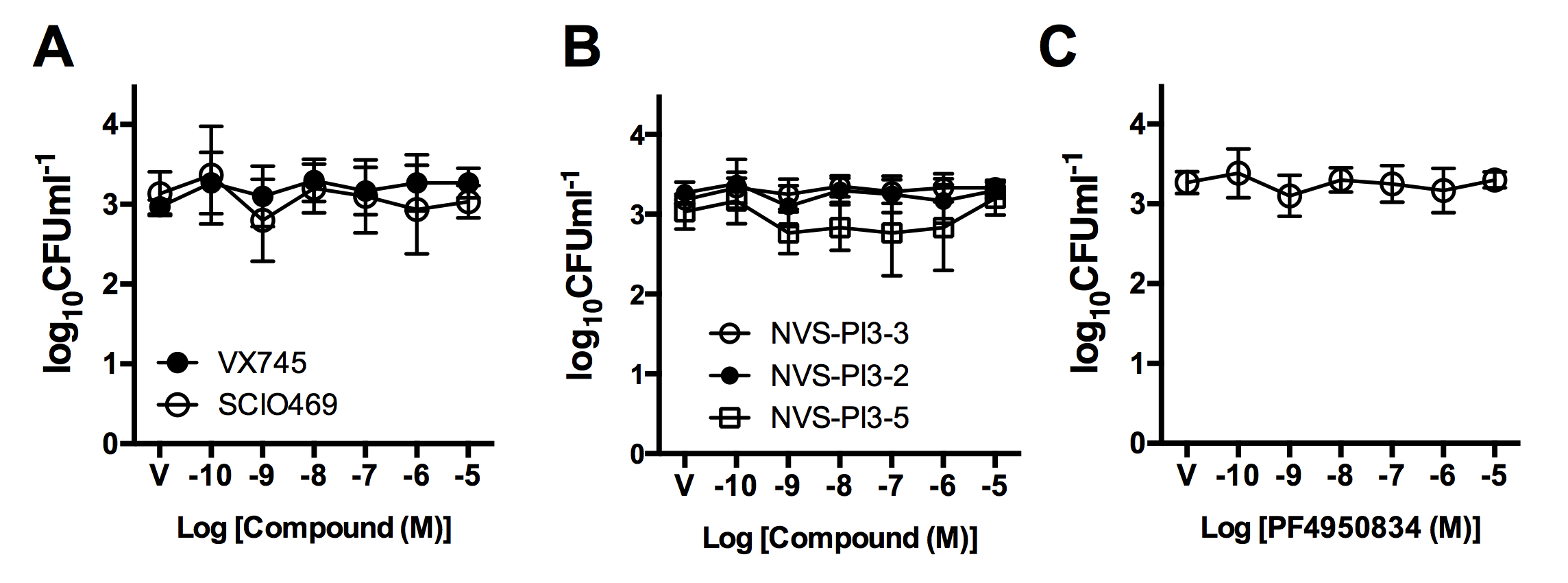

Supplement: S1 Fig — BMDM were treated with vehicle (V) or the designated doses of SCIO469, VX745 (B), NVS-PI3K-2/3/5, (C) or PF4950834 (D) before challenge with Spn at MOI 10. 4 h post challenge, numbers of viable internalized bacteria were determined, n = 3, no significant differences between vehicle and any dose of compound. (TIFF) [file pone.0163139.s001.tiff]

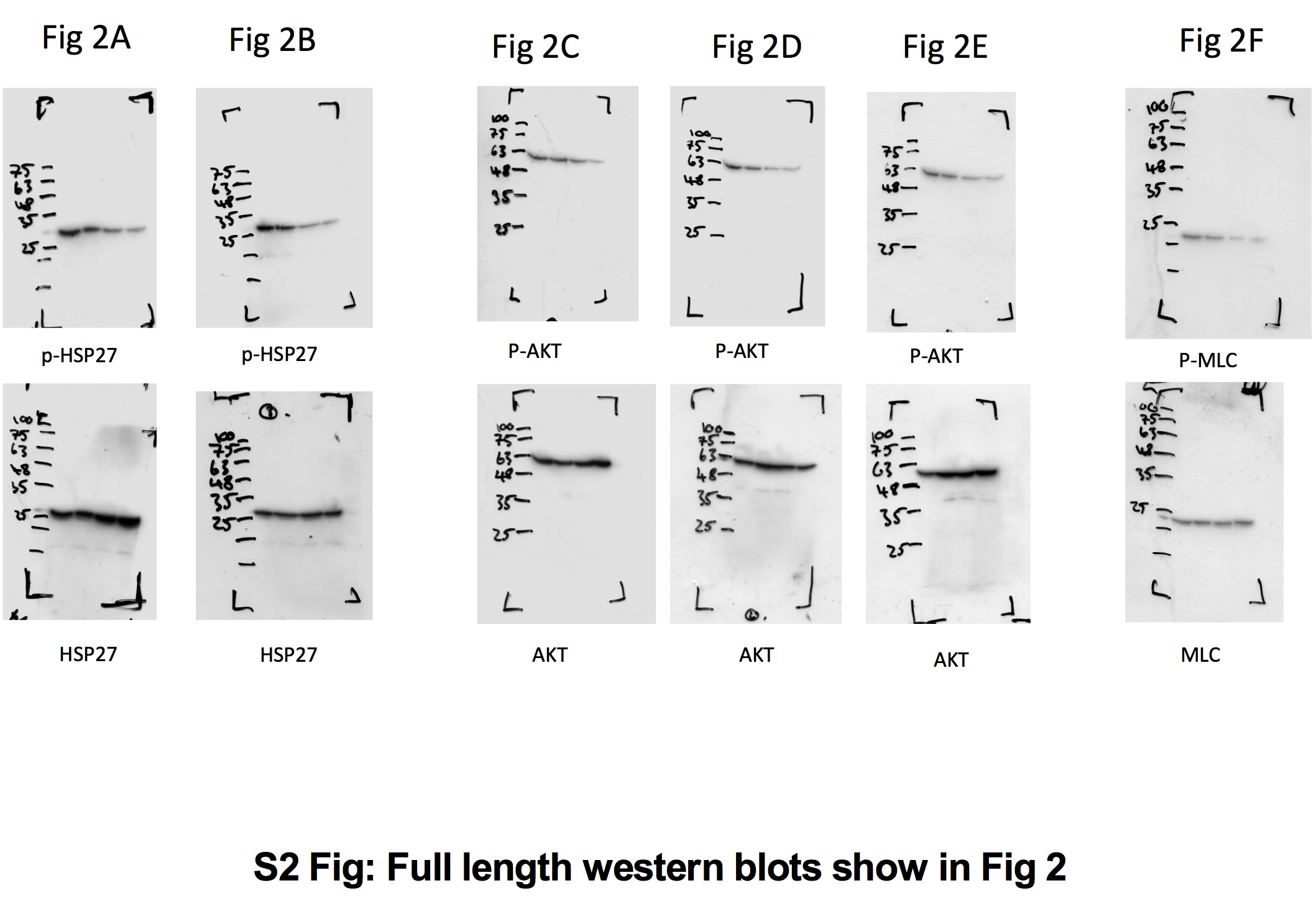

Supplement: S2 Fig — (TIFF) [file pone.0163139.s002.tiff]
